# Supplementary material for: Socio-ecological network structures from process graphs
Source: PLoS One. 2020 Aug 4;15(8):e0232384. doi: 10.1371/journal.pone.0232384 (PMC7402476; doi:10.1371/journal.pone.0232384)
Supplement: S3 Appendix — It contains the pseudocode and brief description of the MSG and SSG algorithms. (PDF) [file pone.0232384.s003.pdf]

This appendix contains a brief description of two algorithms of the P-graph framework: MSG and SSG.

Notations

Let M be a finite non-empty set called as the set of materials. A PNS problem is defined by triplet (P, R, O), where $P(\subset M)$ is the set of products to be produced, $R(\subset M)$ is the set of available raw materials ($P \cap R = \emptyset$), and $O(\subseteq \wp(M) \times \wp(M))$ is the set of operating units, (Cartesian product of the power sets of set M). Let $m \in M$ be a finite set and $o \subseteq \wp(m) \times \wp(m)$. Pair (m, o) defines a P-graph, where the *vertices* of the graph are in set $m \cup o$ and the *arcs* are in set $A = A_1 \cup A_2$ where $A_1 = \{(x, y) | \; y = (\alpha, \beta) \in o \; \& \; x \in \alpha\}$ and $A_2 = \{(y, x) | y = (\alpha, \beta) \in o \; \& \; x \in \beta\}$.

Algorithm MSG

Algorithm MSG [25] generates the maximal structure. It starts from the original P-graph network and removes all materials and operating units that violate any of the five axioms of the framework.

Algorithm MSG is an iterative algorithm with polynomial complexity. The pseudo code of the algorithm is shown below.

Algorithm MSG

Input: sets *P, R, O, M*
Comment: $P \subseteq M, R \subseteq M, O \subseteq \left(\wp(M) \times \wp(M)\right), O \cap M = \emptyset, P \cap R = \emptyset$
Output: *(m,o)*, the maximal structure of synthesis problem *(P,R,O)*, if it exists

---

begin

$O := O \setminus \{(\alpha, \beta) \; | \; (\alpha, \beta) \in O \; \& \; \beta \cap R \neq \emptyset\}$

$M := \bigcup_{(\alpha,\beta) \in O} (\alpha \cup \beta)$

$r := \{x | \; x \in M \setminus R \; \& \; \forall(\alpha, \beta) \in O, x \notin \beta\}$

while $r \neq \emptyset$ **do**

  let $x \in r$

  $M := M \setminus \{x\}$

  $o := \{(\alpha, \beta) \,|\, (\alpha, \beta) \in O \; \& \; x \in \alpha\}$

  $O := O \setminus o$

  $r := (r \cup \{y \,|\, \exists(\alpha, \beta) \in o \text{ such that } y \in \beta \; \& \; \forall(\gamma, \delta) \in O, y \notin \delta\}) \setminus \{x\}$

end while

if $P \nsubseteq M$ **then**

  stop, **comment**: no maximal structure

end if

$p := P, m := \emptyset, o := \emptyset$

while $p \neq \emptyset$ **do**

  let $x \in p$

  $m := m \cup \{x\}$

  $o_x := \{(\alpha, \beta) \,|\, (\alpha, \beta) \in O \; \& \; x \in \beta\}$

  $o := o \cup o_x$

  $p := p \cup \left(\bigcup_{(\alpha,\beta) \in O} \alpha \right) \setminus (R \cup M)$

end while

$$m := \bigcup_{(\alpha,\beta)\in o} (\alpha \cup \beta)$$

end

Algorithm SSG

Algorithm SSG [21] is a recursive algorithm that generates all solution structures (combinatorially feasible networks) included in the maximal structure. The algorithm enumerates all decision mappings based on the set of operating units producing each material (see [21] for details). The pseudo code of the algorithm is shown below.

Algorithm SSG

Input: *P, R, M, o(x)* ($x \in M$)
Comment: *P, R, o(x)* belong to synthesis problem *(P,R,O)*, where $P \subseteq M, R \subseteq M, P \cap R = \emptyset, o(x) = \{(\alpha,\beta) \,|\, (\alpha,\beta) \in O \ \& \ x \in \beta\}, o(x) = \emptyset \Leftrightarrow x \in R$, $\delta[m]$ is a decision mapping on *M*
Output: all solution structures of synthesis problem *(P,R,O)*

---

begin
    if $P = \emptyset$ **then**
        stop, **comment**: there are no solution structures
    end if
    $SSG(P, \emptyset, \emptyset)$
end

procedure $SSG(p, m, \delta[m])$
    if $p = \emptyset$ **then**
        write $\delta[m]$, **comment**: $\delta[m]$ defines a solution structure
        return
    end if
    let $x \in p$
    $C := \wp\big(o(x)\big) \setminus \{\emptyset\}$
    for all $c \in C$ **do**
        if $\forall y \in m, c \cap \big(o(y) \setminus \delta(y)\big) = \emptyset \ \& \ (o(x) \setminus c) \cap \delta(y) = \emptyset$ **then**
            $\delta[m \cup \{x\}] := \delta[m] \cup \{(x, c)\}$
            $SSG\left(p \cup \left(\bigcup_{(\alpha,\beta)\in c} \alpha\right) \setminus (R \cup m \cup \{x\}), m \cup \{x\}, \delta[m \cup \{x\}]\right)$
        end if
    end for
    return
end procedure
