# Supplementary material for: Socio-ecological network structures from process graphs
Source: PLoS One. 2020 Aug 4;15(8):e0232384. doi: 10.1371/journal.pone.0232384 (PMC7402476; doi:10.1371/journal.pone.0232384)
Supplement: S1 Fig — Twenty P-graph structures including MSG & SSG. (PDF) [file pone.0232384.s004.pdf]

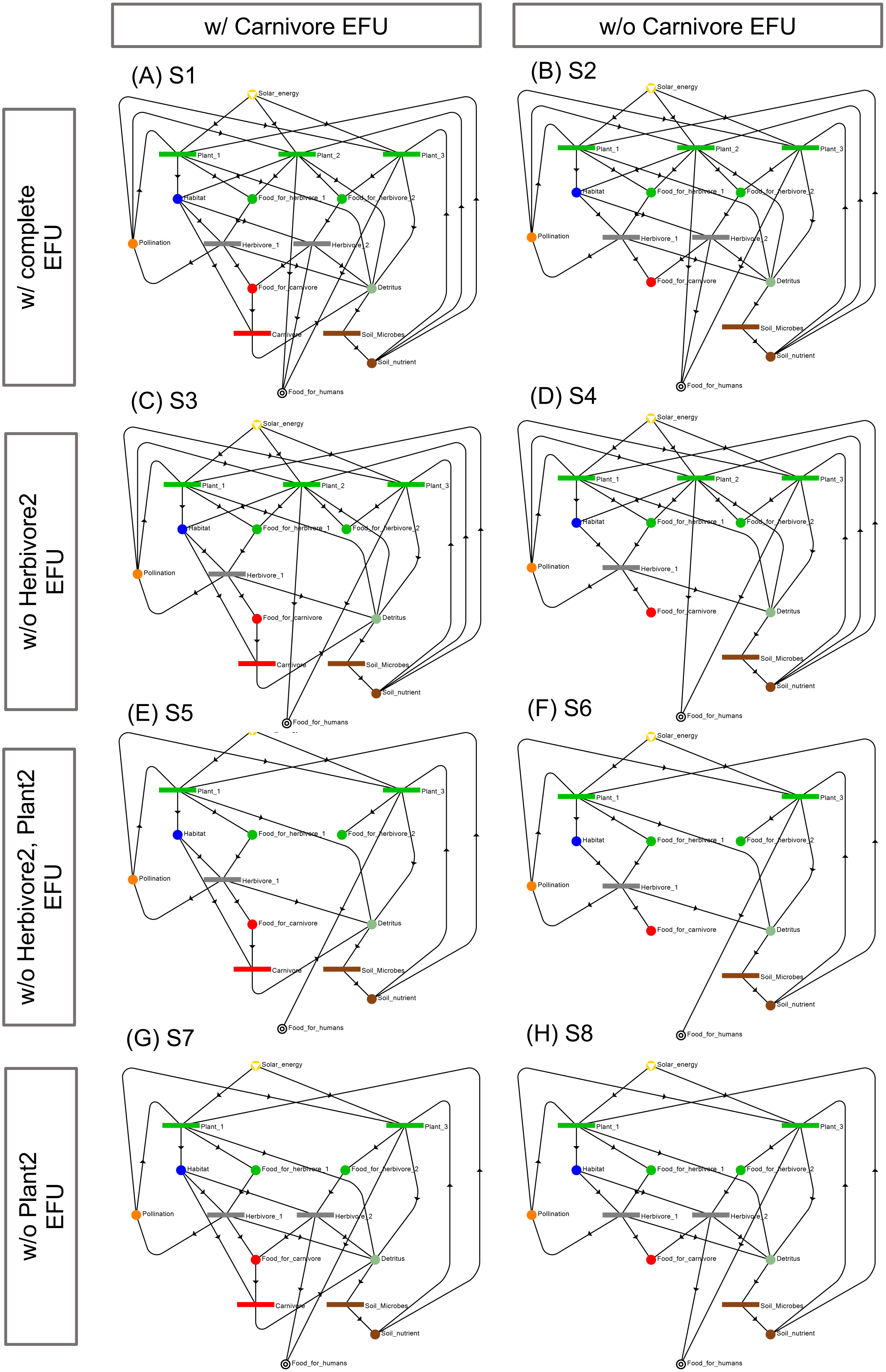

w/ Carnivore EFU
w/o Carnivore EFU
w/ complete EFU
w/o Herbivore2 EFU
w/o Herbivore2, Plant2 EFU
w/o Plant2 EFU
(A) S1
(B) S2
(C) S3
(D) S4
(E) S5
(F) S6
(G) S7
(H) S8
Solar_energy
Plant_1
Plant_2
Plant_3
Habitat
Food_for_herbivore_1
Food_for_herbivore_2
Pollination
Herbivore_1
Herbivore_2
Food_for_carnivore
Detritus
Carnivore
Soil_Microbes
Soil_nutrient
Food_for_humans

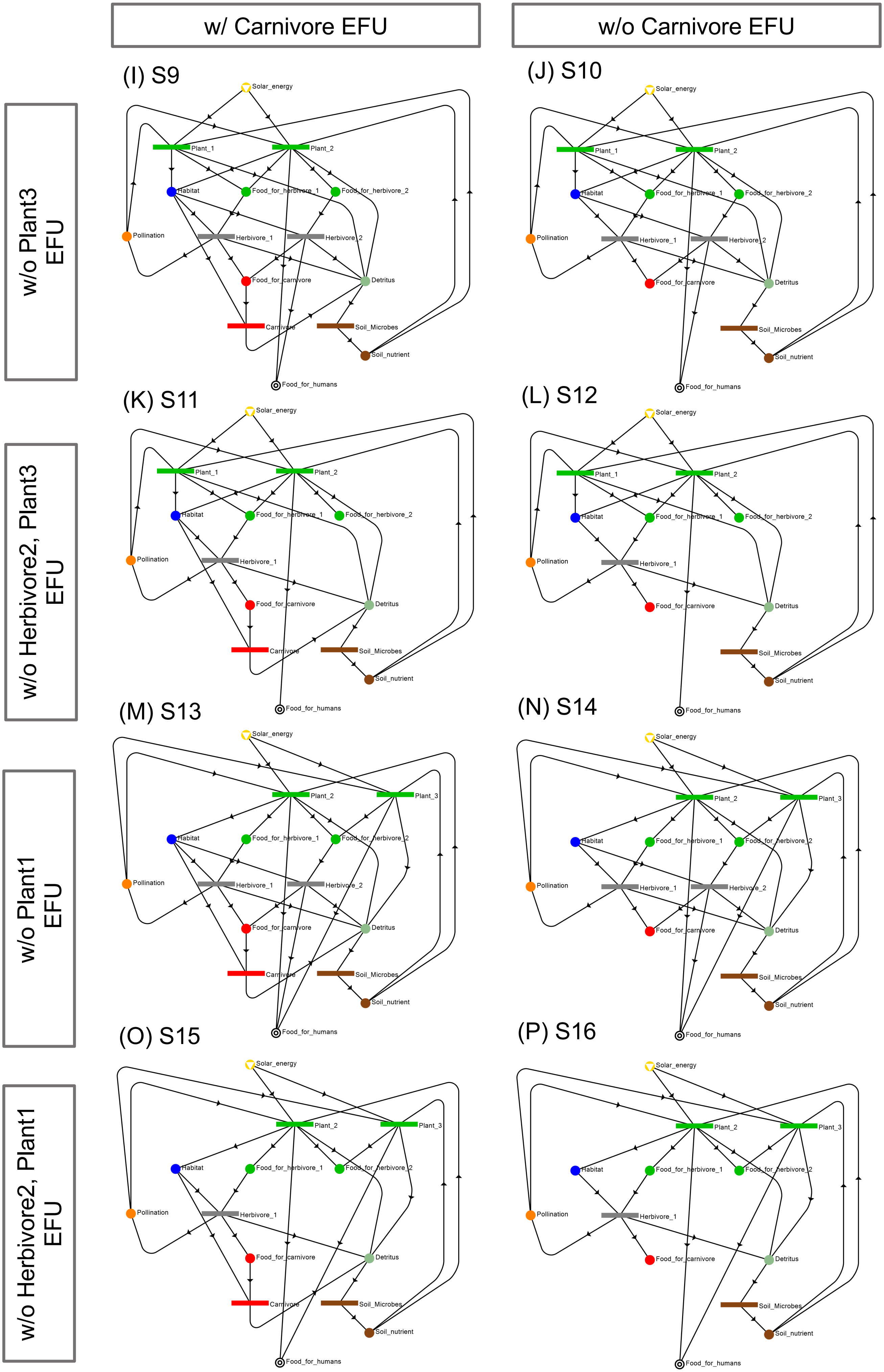

w/ Carnivore EFU
w/o Carnivore EFU
w/o Plant3 EFU
w/o Herbivore2, Plant3 EFU
w/o Plant1 EFU
w/o Herbivore2, Plant1 EFU
(I) S9
(J) S10
(K) S11
(L) S12
(M) S13
(N) S14
(O) S15
(P) S16
Solar_energy
Plant_1
Plant_2
Plant_3
Habitat
Food_for_herbivore_1
Food_for_herbivore_2
Pollination
Herbivore_1
Herbivore_2
Food_for_carnivore
Detritus
Carnivore
Soil_Microbes
Soil_nutrient
Food_for_humans

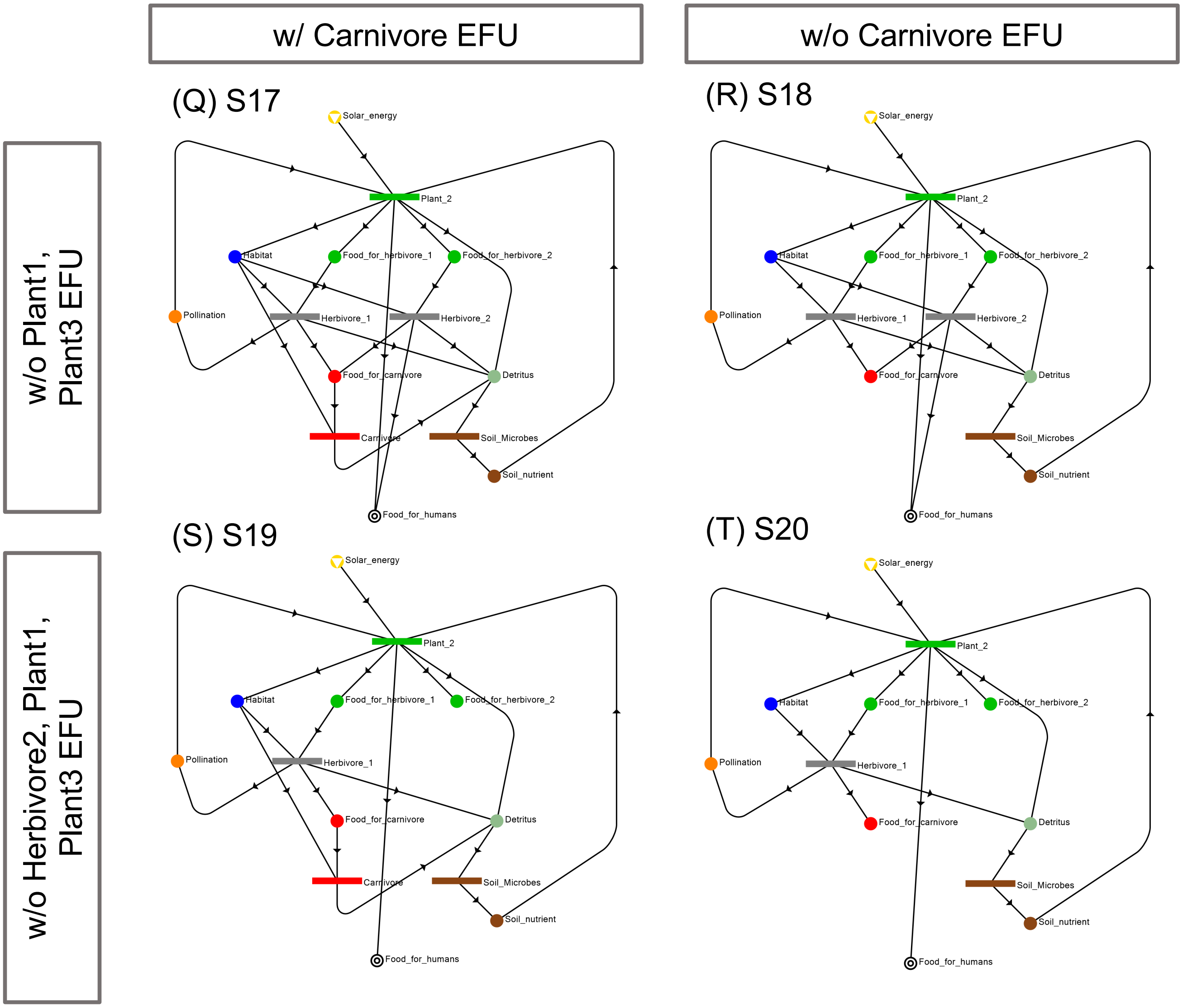

w/ Carnivore EFU
w/o Carnivore EFU
w/o Plant1, Plant3 EFU
w/o Herbivore2, Plant1, Plant3 EFU
(Q) S17
Solar_energy
Plant_2
Habitat
Food_for_herbivore_1
Food_for_herbivore_2
Pollination
Herbivore_1
Herbivore_2
Food_for_carnivore
Detritus
Carnivore
Soil_Microbes
Soil_nutrient
Food_for_humans
(R) S18
Solar_energy
Plant_2
Habitat
Food_for_herbivore_1
Food_for_herbivore_2
Pollination
Herbivore_1
Herbivore_2
Food_for_carnivore
Detritus
Soil_Microbes
Soil_nutrient
Food_for_humans
(S) S19
Solar_energy
Plant_2
Habitat
Food_for_herbivore_1
Food_for_herbivore_2
Pollination
Herbivore_1
Food_for_carnivore
Detritus
Carnivore
Soil_Microbes
Soil_nutrient
Food_for_humans
(T) S20
Solar_energy
Plant_2
Habitat
Food_for_herbivore_1
Food_for_herbivore_2
Pollination
Herbivore_1
Food_for_carnivore
Detritus
Soil_Microbes
Soil_nutrient
Food_for_humans
