# Supplementary material for: Socio-ecological network structures from process graphs
Source: PLoS One. 2020 Aug 4;15(8):e0232384. doi: 10.1371/journal.pone.0232384 (PMC7402476; doi:10.1371/journal.pone.0232384)
Supplement: Attachment — Submitted filename: Rebuttal for PONE-D-20-10193.pdf [file pone.0232384.s005.pdf]

Dr. Joerg Heber
Editor-in-chief
PLOS One

Re: Revision of manuscript PONE-D-20-10193

Dear Editor-in-Chief Dr. Heber,

Please find enclosed the revised version of our manuscript entitled "Socio-Ecological Network Structures from Process Graphs".

Our general assessment is that most of the criticisms (especially from Reviewer 2) arise from a lack of a clear understanding of the capabilities and limitations of the P-graph framework. Thus, we have revised the manuscript to give a clearer picture of the class of problems that our work addresses via P-graph. We also discuss the possible interface of P-graph with other more well-known tools.

We strongly believe that we have successfully addressed all concerns raised by the reviewers and we hope that you will accept this manuscript for publication in PLOS One.

Sincerely,

Angelyn Lao, Heriberto Cabezas, Ferenc Friedler, Ákos Orosz, and Raymond R. Tan

POINT-BY-POINT RESPONSE

(text in red pertains to the comments of the reviewers, the response is in black)

Comment from the Editor:

Reviewers clearly disagree in their assessment. Please address the comments to the extent possible. Also, interconnectedness of socio-ecological systems is discussed in the field of resilience, especially in papers that view system resilience as property of interconnected networks (e.g. Ganin et al., (2016). Operational resilience: concepts, design and analysis. *Nature Scientific Reports,* **6(1); Linkov, I., & Trump, B. D. (2019).** ***The Science and Practice of Resilience.*** **Springer, Amsterdam.).** Discussion of these issues may be helpful for positioning the paper.

Response:

Following the advice of the reviewer, we included a discussion in relation to resilience and cited the suggested references. We would like to thank the editor for the recommendation because it provides additional insights on how P-graph can be used in future work. We have added relevant discussion of such prospects at the end of the revised paper.

> In addition to its stand-alone capabilities, the P-graph framework can also be used in conjunction with other concepts and tools that are relevant to the effective management of ecosystems. Biodiversity loss and ecosystem collapse are major environmental issues; understanding emergent properties of ecological networks can provide valuable insights for retarding or stopping irreversible damage. The concept of resilience has become an important aspect in the design of different man-made networks which need to be designed to withstand shocks [32]. Resilient systems need to be planned to be able to absorb, recover from, and adapt to perturbations at the network level [33]. The concept can be extended to managing ecosystems to allow them to withstand pressure from climate change, human encroachment, and disruptive events such as natural disasters. In PNS problems, P-graph methodology has been shown to be effective for designing robust and resilient systems via process-level redundancy and network level degeneracy [34]. This capability can also be readily extended to PNS-like problems in ENA, so that rational and effective plans can be developed for the protection of ecosystems.

REFERENCES:

32. Linkov I, Trump BD. The science and practice of resilience. Springer International Publishing; 2019.

33. Ganin AA, Massaro E, Gutfraind A, Steen N, Keisler JM, Kott A, Mangoubi R, Linkov I. Operational resilience: concepts, design and analysis. Sci Rep. 2016 Jan;6(1):1-2

Comment from Reviewer 1:

This is a paper suggesting an alternative approach to ecological network analysis (ENA), namely the process graph (or P-graph) one. The authors draw this methodology from the industrial applications' field and argue that it is superior to traditional ENA as it separates every operational and intermediate component of such a network explicitly and gives insights of its viable realizations out of all its possible ones. More importantly, it has a say on the critical components of such systems, thus elucidating managing aspects of those systems. Lastly, the framework is neatly presented in a generic example rising from first principle, ecological arguments. The example is reproducible with code provided in Visual Basic and Excel macros. The work is coherent and consistent with the established methods of P-graphs and has value to at least the audience of ecology. I am therefore confident that it is suitable for publication in PLOS One and will be an asset to the community. As some thoughts and secondary remarks however, I would advise the authors to consider the following points prior to finalising the publication of this paper:

1.1. Petri nets seem to have a lot of common properties with P-graphs. What is the connection of ecological P-graphs to Petri nets? How are the two different? Broaching this topic in the text would broaden the work's scope.

Response:
We agree with the reviewer. In fact, Lakner *et al.* (2017) discussed the main similarities and differences between Petri nets and P-graphs. P-graph methodology is mainly used for automated assembly of networks from predefined building blocks, while Petri nets are used to simulate dynamic behavior of predefined networks. They also showed how to efficiently combine the application of these two approaches. Following the advice of the reviewer, we added the following discussion in our manuscript.

In [15], P-graph and Petri nets are compared and combined. They showed the correspondence between P-graph and Petri nets, and applied the algorithms (MSG and SSG) to P-graphs converted from Petri nets. In the hybrid P-graph and Petri net methodology, the study illustrates the potential of P-graph to be used in conjunction with other mainstream modelling techniques. Although these two approaches have been developed for different purposes, they both use the same structural representation - bipartite directed graphs. The vertices of P-graph are the elements of $V = M \cup O$, where materials (M) and operating units (O) are disjoint sets. The nodes of a Petri net are the elements of $N = P \cup T$, where places (P) and transitions (T) are disjoint sets. Operating units and materials of P-graph correspond to transitions and places that are defined in a Petri net. The reason for representing them using a bipartite graph is, however, rather different. Despite the similarities between P-graph and Petri nets, the target of application of these two approaches are different. P-graphs optimize the desired targets of the process systems using the available resources. P-graphs also generate the optimal structure and optimal parameter values of process systems. P-graphs are applied to steady state processing systems. In contrast, Petri nets are applied primarily for modeling concurrent and asynchronous distributed dynamical systems.

REFERENCE

[15] Lakner R, Friedler F, Bertók B. Synthesis and analysis of process networks by joint application of P-graphs and Petri nets. International Conference on Application and Theory of Petri Nets and Concurrency 2017 Jun 25 (pp. 309-329). Springer, Cham.

1.2. It is implicit but poorly described in the materials and methods section that the Accelerated-Branch-and-Bound (ABB) algorithm comes into the analysis flow and process information, which, in a 3-level procedure, makes the application of the scheme MSG-→SSG-→ABB for ecological networks sensible.

Response:
The current work has not gone to the extent of ABB, which we will address in future work. Our main focus at this stage is to address the problem of rigorously deducing candidate ecosystem structures from the best available scientific knowledge on the properties of the components. We have improved the discussion of the case study to further emphasize this point.

The MSG algorithm rigorously generates a network which is the union of all structurally or combinatorially feasible networks that can be generated from the component units [25]. The SSG is capable of generating all combinatorially feasible networks, based on total enumeration of possible structures arising from localized relationships between materials and processes or services and functional units in the case of ecosystems [22]. Combinatorial feasibility is based solely on network connectivity and does not account for stream flow rates or quantities. The accelerated branch-and-bound algorithm (ABB) can then be used to effectively determine optimal and near-optimal solutions to PNS problems by evaluation of the performance of candidate networks, while excluding infeasible and redundant solutions from the search strategy. This effectiveness is derived from the use of implicit information embedded in all PNS problems [26]. A recent review paper gives a comprehensive survey of P-graph literature focusing on engineering applications [23]. The potential applicability of this framework to deal with PNS-like problems in generalized networks – such as economic systems or organizational structures – is discussed by [27]. In this study, the focus is purely on structural aspects and does not delve into stream flow rates.

1.3. Since this is a recurrent theme throughout the paper, an example or a procedural (or algorithmic) explanation of the "local interactions" giving rise to the MSG would be useful for the reader who is not familiar with the algorithm. Especially illustrative would be to draw a connection of the local interactions with an ecosystem's example.

Response:
Following the advice of the reviewer, we added an appendix (see "S3 Appendix. Brief introduction of MSG and SSG algorithms.") with a very brief tutorial of the MSG and SSG algorithms. It includes the pseudocode and brief description of the algorithms.

Algorithm MSG

Algorithm MSG [25] generates the maximal structure. It starts from the original P-graph network and removes all materials and operating units that violate any of the five axioms of the framework.

Algorithm MSG is an iterative algorithm with polynomial complexity. The pseudo code of the algorithm is shown below.

Algorithm MSG

Input: sets *P, R, O, M*
Comment: $P \subseteq M, R \subseteq M, O \subseteq (\wp(M) \times \wp(M)), O \cap M = \emptyset, P \cap R = \emptyset$
Output: *(m,o)*, the maximal structure of synthesis problem *(P,R,O)*, if it exists

---

begin
    $O := O \setminus \{(\alpha,\beta) \mid (\alpha,\beta) \in O \ \& \ \beta \cap R \neq \emptyset\}$
    $M := \bigcup_{(\alpha,\beta)\in O} (\alpha \cup \beta)$
    $r := \{x \mid x \in M \setminus R \ \& \ \forall(\alpha,\beta) \in O, x \notin \beta\}$
    while $r \neq \emptyset$ **do**
        let $x \in r$
        $M := M \setminus \{x\}$
        $o := \{(\alpha,\beta) \mid (\alpha,\beta) \in O \ \& \ x \in \alpha\}$
        $O := O \setminus o$
        $r := (r \cup \{y \mid \exists(\alpha,\beta) \in o \text{ such that } y \in \beta \ \& \ \forall(\gamma,\delta) \in O, y \notin \delta\}) \setminus \{x\}$
    end while
    if $P \nsubseteq M$ **then**
        stop, comment: no maximal structure
    end if
    $p := P, m := \emptyset, o := \emptyset$
    while $p \neq \emptyset$ **do**
        let $x \in p$
        $m := m \cup \{x\}$
        $o_x := \{(\alpha,\beta) \mid (\alpha,\beta) \in O \ \& \ x \in \beta\}$
        $o := o \cup o_x$
        $p := p \cup \left(\bigcup_{(\alpha,\beta)\in O} \alpha \right) \setminus (R \cup M)$
    end while
    $m := \bigcup_{(\alpha,\beta)\in o} (\alpha \cup \beta)$
end

Algorithm SSG

Algorithm SSG [21] is a recursive algorithm that generates all solution structures (combinatorially feasible networks) included in the maximal structure. The algorithm enumerates all decision mappings based on the set of operating units producing each material (see [21] for details). The pseudo code of the algorithm is shown below.

Algorithm SSG

Input: *P, R, M, o(x)* $(x \in M)$
Comment: *P, R, o(x)* belong to synthesis problem *(P,R,O)*, where $P \subseteq M, R \subseteq M, P \cap R = \emptyset, o(x) = \{(\alpha,\beta) \mid (\alpha,\beta) \in O \ \& \ x \in \beta\}, o(x) = \emptyset \Leftrightarrow x \in R$, $\delta[m]$ is a decision mapping on *M*
Output: all solution structures of synthesis problem *(P,R,O)*

---

begin
    if $P = \emptyset$ **then**
        stop, comment: there are no solution structures
    end if
    $SSG(P, \emptyset, \emptyset)$
end

procedure $SSG(p, m, \delta[m])$
    if $p = \emptyset$ **then**
        write $\delta[m]$, **comment**: $\delta[m]$ defines a solution structure
        return
    end if
    let $x \in p$
    $C := \wp(o(x)) \setminus \{\emptyset\}$
    for all $c \in C$ **do**
        if $\forall y \in m, c \cap (o(y) \setminus \delta(y)) = \emptyset \ \& \ (o(x) \setminus c) \cap \delta(y) = \emptyset$ **then**
            $\delta[m \cup \{x\}] := \delta[m] \cup \{(x,c)\}$
            $SSG(p \cup \left(\bigcup_{(\alpha,\beta)\in c} \alpha \right) \setminus (R \cup m \cup \{x\}), m \cup \{x\}, \delta[m \cup \{x\}])$
        end if
    end for
    return
end procedure

We illustrated the local interactions of and the role played by each component in the ecosystem in Figure 3. It shows the ecosystem components prior to assembly into the maximal structure. This is the "network assembly" capability of P-graph.

A socio-ecological system can be visualized as shown in Fig 2. In practice, the network representing such a system needs to be deduced from the local properties of its components or building blocks, as shown in Fig 3. In this system, we have defined specific characteristics for the plants and herbivores. For the plants, Plant 1 provides food for Herbivore 1, Habitat for herbivores and carnivores, and nutrients for Detritus (Fig 3A); Plant 2 not only provides food for Herbivore 1, Herbivore 2, and Humans, but also contributes to the Detritus and Habitat (Fig 3B); and Plant 3 contributes to the Detritus, and provides food for Herbivore 2 and Humans (Fig 3C). As for herbivores, both Herbivore 1 (Fig 3F) and Herbivore 2 (Fig 3G) contribute to the Detritus and provide food for Carnivore. Only Herbivore 1 contributes to the pollination, and only Herbivore 2 provides food for humans.

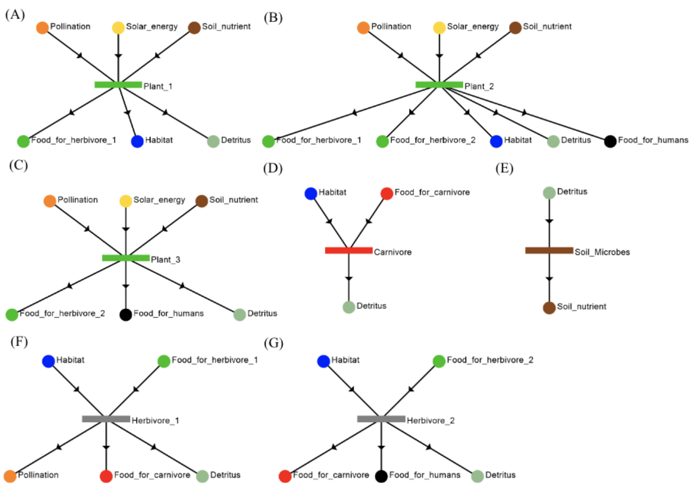

Fig 3. Local interactions of the ecosystem components. The ecosystem functional units presented here also corresponds to those listed in Table 2. It includes `Plant1` (A), `Plant2` (B), `Plant3` (C), `Carnivore` (D), `Soil_microbes` (E), `Herbivore1`(F), and `Herbivore2` (G).

1.4. The way the numerator of the criticality index is explained might cause some confusion as to whether the systems referred to are viable or not. I would add this explicitly.

Response:
To avoid confusion on what the numerator of the critical index is, we have improved Table 2 by including the number of alternate structures containing functional component i (denoted by N_i) and their corresponding criticality index (denoted by IC_i).

Table 2. The ecosystem functional units contained in each of the 20 structures.

Structure Label	Plant1 Fig 3A	Plant2 Fig 3B	Plant3 Fig 3C	Herbivore1 Fig 3F	Herbivore2 Fig 3G	Carnivore Fig 3D	Soil microbes Fig 3E
S1	✓	✓	✓	✓	✓	✓	✓
S2	✓	✓	✓	✓	✓		✓
S3	✓	✓	✓	✓		✓	✓
S4	✓	✓	✓	✓			✓
S5	✓		✓	✓		✓	✓
S6	✓		✓	✓			✓
S7	✓		✓	✓	✓	✓	✓
S8	✓		✓	✓	✓		✓
S9	✓	✓		✓	✓	✓	✓
S10	✓	✓		✓	✓		✓
S11	✓	✓		✓		✓	✓
S12	✓	✓		✓			✓
S13		✓	✓	✓	✓	✓	✓
S14		✓	✓	✓	✓		✓
S15		✓	✓	✓		✓	✓
S16		✓	✓	✓			✓
S17		✓		✓	✓	✓	✓
S18		✓		✓	✓		✓
S19		✓		✓		✓	✓
S20		✓		✓			✓
N_i	12	16	12	20	10	10	20
IC_i	0.6	0.8	0.6	1	0.5	0.5	1

1.5. As specified in the text, plant 2 is necessary to ensure the "food for the humans" terminal. This is owed to the weave of interconnections following the intermediate operational units and nodes as resulting from the original assignment of the system's components to O and M nodes and the P-graph's algorithms. I find this to be a remarkable result with lots of extensions for practical ecosystem applications and would advise it to be further elaborated and highlighted.

Response:
We added a subsection "Implications and Prospects" to elaborate the point mentioned by the reviewer, both for the case study, and for the generalization of the principle.

1.6. Although nothing significant through a superficial read, there are some minimal language slips and therefore a proof-reading would eliminate the few issues standing out.

Response: We have carefully proof-read the manuscript and corrected grammatical errors and issues that we thought might stand out.

Comment from Reviewer 2:

The authors' manuscript applies the process graph (P-graph) approach from industrial plant design to ecological systems. The ecological application of the P-graph is proposed as a bipartite representation of an ecosystem in which processes and ecosystem compartments are two distinct parts in the network. All compartments are "indirectly" connected via processes. The authors' state that the P-graph approach has several advantages over current ecological network modeling approaches. The main being that the use of the bipartite (process-compartment) structure, permits compartments to have multiple roles in the ecosystem, such as both prey and pollinator. The majority of ENA models only consist of a single "currency", typically biomass or nutrients. The authors also present how the identification of "maximal" and "minimal" network structures, relative to a desired output from the ecosystem, permits the quantification of the effects of species loss, efficacy of reconstruction efforts and maximum sustainable exploitation by humans via the calculation of a criticality index. The authors present an application of the method to a "stylized" example network and conclude that the P-graph method is a novel approach that can provide a useful tool for solving ecological optimization problems in the context of increasing global pressures on the environment.

2.1. My primary concern is that the P-graph method appears to be a special case of a multi-layer graph approach, which has already been published widely in ecology. As such, I do not see the claims of novelty justified without a more complete graph theoretic analysis of the P-graph method as it compares to multi-layer networks. In particular, two high profile articles have already been published that provide developments in this area that are not cited:

Pilosof, S., Porter, M., Pascual, M. et al. The multilayer nature of ecological networks. Nat Ecol Evol 1, 0101 (2017).

Matthew C. Hutchinson Bernat Bramon Mora Shai Pilosof Allison K. Barner Sonia Kéfi Elisa Thébault Pedro Jordano Daniel B. Stouffer Seeing the forest for the trees: Putting multilayer networks to work for community ecology. Func. Ecol. 33, 2 (2018).

Response:
We understand the concern of the reviewer. In the revised paper, we explain the concept of P-graphs more clearly to address points that were misunderstood by the reviewer. For this reason, we made further elaborations on P-graphs and added some discussions on the differences between multilayer networks and P-graphs. The key feature of P-graph which is relevant to ecological network analysis is the rigorous, automated assembly of candidate network structures based on the best available localized scientific knowledge on the system components. By comparison, a multilayer network focuses on analysis of the behavior of systems with a priori structures. This makes the target applications of these two approaches different. Although there are differences between the two approaches, we agree with the reviewer and also suspect that it is possible to convert multilayer graphs to P-graphs (and vice versa). In [15], they showed the correspondence between P-graph and Petri nets, and applied the optimization algorithms (MSG and SSG) to P-graphs converted from Petri nets. To our knowledge, there is no similar study done for P-graphs and multilayer networks. This can be proposed as future work.

Discussion and comparison of multilayer network to P-graph are inserted in the Introduction and Discussion section:

Multilayer networks emerge as a framework to study complex ecological systems that account for multiple interaction types and multiple layers of complexity, such as the heterogeneous nature of the systems. In an ecological context, different network layers commonly represent different types of interactions, different communities of species, different points in time, and so on [16, 17]. A multilayer graph $G_M = (V_M, E_M)$ contains set of nodes V_M that is represented by $V \times L_1 \times \cdots \times L_d$ with layers L_i where $i = 1, \cdots, d$; and a set of edges E_M that is defined by $V_M \times V_M$. Unlike P-graph that is mainly applied to steady state processing systems and optimize desired targets of the process systems, multilayer networks approach is mainly applied for modeling complex dynamical systems. Even though multilayer networks have been widely applied in ecology, there is a huge potential in developing the applications of P-graphs in ecology for different target approaches. To our knowledge, there is no correspondence study done for P-graphs and multilayer networks.

:

be formed from a set of building blocks (i.e., species or compartments). Thus, if the equivalence between P-graphs and multiplex graphs can be rigorously established in the future, then the MSG and SSG algorithms can also be used to generate multilayer networks based on knowledge of the local interactions of each species. This possibility is intriguing, but remains speculative at this stage. Further research is needed to establish if the two mapping approaches are indeed equivalent.

REFERENCES

15. Lakner R, Friedler F, Bertók B. Synthesis and analysis of process networks by joint application of P-graphs and Petri nets. International Conference on Application and Theory of Petri Nets and Concurrency 2017 Jun 25 (pp. 309-329). Springer, Cham.

16. Pilosof S, Porter MA, Pascual M, Kéfi S. The multilayer nature of ecological networks. Nature Ecology & Evolution. 2017 Mar 23;1(4):1-9.

17. Hutchinson MC, Bramon Mora B, Pilosof S, Barner AK, Kéfi S, Thébault E, Jordano P, Stouffer DB. Seeing the forest for the trees: Putting multilayer networks to work for community ecology. Functional ecology. 2019 Feb;33(2):206-17.

2.2. One specific criticism is that the P-graph approach and associated metrics, as currently described, assume an unweighted graph, which is a major step back from the multi-layer graph methods that already incorporate weighted and signed edge values.

Response: In our work, establishing direct and indirect connectivity regardless of weight is taken as Step 1. This initial step is necessary since network topology or structure often has a larger effect on system characteristics than variations within any given structure. With this said, the P-graph approach also incorporates weighted arcs pertaining to flow rates and represented in an incidence “flow” matrix. However, the analysis shown in this study focuses purely on structural aspects and does not delve into stream flow rates.

2.3. In addition, multilayer analyses have been implemented in python and are available across all major operating systems that run Python, as opposed to p-graph, which is only available on Windows.

Response: It is important to distinguish the algorithm from its software implementation. We acknowledge the current limitations of P-graph Studio (P-graph software) which is configured for engineering design problems. Addressing the issues with respect to software compatibility have been included in our future plan. The VBA code included in the appendix represents an initial step in this direction; future implementation in Python is possible in principle. Currently, our focus is on extending the applications of P-graph to other fields of study, and attracts talented researchers to join us in the conceptual/theoretical innovations and developments of P-graphs.

2.4. Within the context of P-graph and how it is presented, the MSG and SSG algorithms are not described and I didn't see references given that describe them. As such I have indicated above that not all data have been provided, as this is a core component of the paper. Where they are defined in the text, the citation is for a general ENA modeling method by Fath et al. that doesn't discuss these algorithms. This should be corrected by either adding such a reference or including a description of the algorithms here.

Response:
Following the advice of the reviewer, we added an appendix (see “S3 Appendix. Brief introduction of MSG and SSG algorithms.”) with a very brief tutorial of the MSG and SSG algorithms. It includes the pseudocode and brief description of the algorithms. The development and detailed discussion of these algorithms can be found in [20,21] which we have already cited. We also cited [24], a reader-friendly online tutorial by the developers of P-graph Studio.

20. Friedler FE, Tarjan K, Huang YW, Fan LT. Combinatorial algorithms for process synthesis. Comput Chem Eng. 1992 May 1;16:S313-20.
21. Friedler F, Tarjan K, Huang YW, Fan LT. Graph-theoretic approach to process synthesis: Axioms and theorems. Chem Eng Sci. 1992 Jun 1;47(8):1973-88.
24. P-Graph [Internet]. [cited 2020 Feb 10]. Available from: http://p-graph.com/

2.5. The MSG method assumes the described P-graph as the maximal network; however, many ecosystems of interest are in a state of human induced disturbance and may represent a reduced state of the ecosystem, as is acknowledged by the authors. How can such assumptions about the maximal network be accounted for when applying such analyses to the management of real ecosystems?

Response: The maximal structure is a mapping tool, and it need not represent the true ecological network. It is the union of all structurally feasible networks that a set of components can form. Thus, the true network that the researcher seeks to deduce is a subset of this maximal structure. These subsets can also be enumerated comprehensively using the second algorithm, SSG. We would also like to clarify that a maximal network does not imply that it is a good ecosystem. We have included Figure 3 to further illustrate the local interactions of the ecosystem functional units that are composed in a maximal structure.

A socio-ecological system can be visualized as shown in Fig 2. In practice, the network representing such a system needs to be deduced from the local properties of its components or building blocks, as shown in Fig 3. In this system, we have defined specific characteristics for the plants and herbivores. For the plants, Plant 1 provides food for Herbivore 1, Habitat for herbivores and carnivores, and nutrients for Detritus (Fig 3A); Plant 2 not only provides food for Herbivore 1, Herbivore 2, and Humans, but also contributes to the Detritus and Habitat (Fig 3B); and Plant 3 contributes to the Detritus, and provides food for Herbivore 2 and Humans (Fig 3C). As for herbivores, both Herbivore 1 (Fig 3F) and Herbivore 2 (Fig 3G) contribute to the Detritus and provide food for Carnivore. Only Herbivore 1 contributes to the pollination, and only Herbivore 2 provides food for humans.

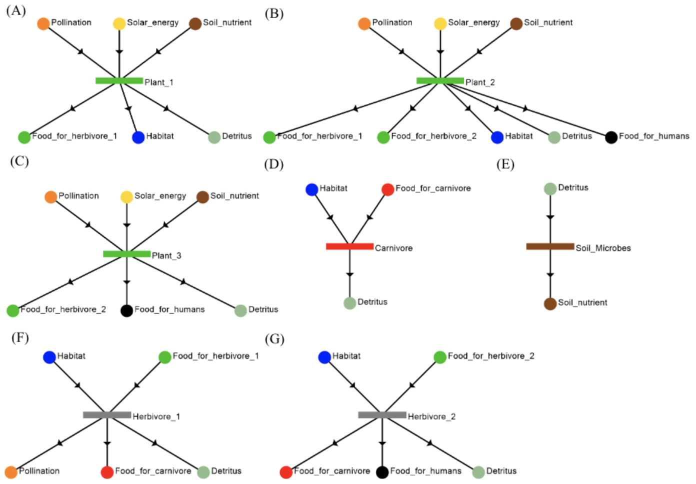

Fig 3. Local interactions of the ecosystem components. The ecosystem functional units presented here also corresponds to those listed in Table 2. It includes `Plant1` (A), `Plant2` (B), `Plant3` (C), `Carnivore` (D), `Soil_microbes` (E), `Herbivore1`(F), and `Herbivore2` (G).

2.6. Every ecosystem functional unit is well defined as an axiom of the p-graph method. This would be possible in a controlled production setting of a factory but is unrealistic in the vast majority of ecosystems both due to the numerous functions and because of behavioral and evolutionary variability. Given these as potential issues, what is the consequence for the P-graph approach?

Response: From observations we create an abstraction in the form models which may be explicit or implicit. The definition of a functional unit is a model for the behavior of a species observed based on observations. If the observations accurately represent reality, and if these are abstracted into a model accurately, then the resulting model represents reality with fidelity. In this study, the goal is to deduce structurally feasible ecosystem networks from the best available scientific data on the components. Even the use of established ENA tools such as single- or multi-layer networks relies on the latter assumption. Incorrectly specified building blocks will lead to erroneous system-level results regardless of which methodology is used. Network features will of course change if the local properties of an ecosystem compartment or species change due to the discovery of new scientific knowledge (e.g., consider, for example, if a species thought by zoologists to be a carnivore is later found to be an omnivore). Once the underlying assumptions about the system components change, adjusted networks can be deduced using the network assembly capability of P-graph.

2.7. There is a discussion point that states a product of the analysis being that given no direct link from carnivores to human food, carnivores would be determined to be non-essential. This seems to point to an issue with this approach and how it is not accounting for indirect effects. For example, what is the indirect effect of carnivores on human food production via the nutrient inputs from carnivores both directly and indirectly through predation? Perhaps this is an issue with interpretation of the analysis, but, if this were used as a management tool, under this interpretation, carnivores might be incorrectly deemed lower in importance relative to human food production than they are in actuality.

Response:
The influence of the carnivore as indicated by the reviewer can certainly be included in eth analysis. That was not done here because the ecosystem is meant to illustrate the use of the P-graph in generating the ecosystem structure from knowledge of the component species. We do not claim that this illustrative ecosystem is an accurate representation of a real ecosystem. Any indirect effects can automatically be deduced via P-graph, provided that the local interactions of each system component is specified as completely as possible based on current scientific knowledge. It should be noted that in this case, the hypothetical carnivore is not essential from the utilitarian perspective as the case study assumes that food is the only ecosystem service that is specified. Our example assumes a primitive, subsistence-level hunter gatherer society. In principle, it is possible to include a range of tangible and intangible ecosystem services as well. For example, “aesthetic satisfaction” in the existence of species that may have no known, tangible utilitarian value to humans can be included in a P-graph model by representing this value explicitly as a product stream. The use of this modelling framework is predicated on the correct specification

of ecosystem components. Incorrect specification of these components can lead to a network that does not accurately represent the real ecosystem. However, it should be noted that the same consequence will result from the use of any other conventional ENA tool using incorrect initial assumptions.

2.8. With regard to the criticality index, there is a significant limitation resulting from the lack of a weighted metric in that sufficient quantities required for functions are not quantified. The un-weighted graph assumes equivalence and implies sufficiency to meet the requirements of each function. Continuing with the carnivore example, in a real ecosystem as a sequential extirpation of prey species were to proceed the availability of prey would eventually reach a point at which there would be an insufficient amount to sustain a viable population of carnivores. That level might be reached prior to having lost all herbivore species, especially if the abundances of herbivores is uneven, as is often the case in ecosystems. This and similar issues require greater clarification prior to recommending such a tool as a useful resource for ecosystem management decisions.

Response: The purpose of the proposed index is to provide a metric that is based purely on topological properties of the deduced ecological network. It represents the structural importance of species. Whether any ecosystem structure can yield a dynamically viable ecosystem is a separate question. The index provides baseline information even before the magnitudes of flows and interactions are incorporated into the model. Thus, the set of structurally feasible networks enumerated via P-graph acts as an upper bound; the set of sustainable ecosystems, when taking into account viable populations of species, will be a subset of this list of networks. The analysis sequence is: (1) structure, (2) dynamics, (3) management recommendations. Our structural index, however, does say some important things like: the system cannot operate without soil microbes or herbivores.

2.9. Given that society is only a final consumer in the example models, and is strongly implied in the list of P-graph axioms, I struggle to see how the term socio-ecological applies to this approach as represented here. Does the "socio-" in socio-ecological refer to human society here? If so, does the P-graph approach have greater potential for integrating more dimensions of human interactions with ecosystems? If not, is "socio-" referring to the "sociological" nature of non-trophic interactions (e.g. pollination)?

Response:
Our work focuses on the analysis of ecosystems as a source of ecosystem services for an external human society that acts as a “customer” or exploiter. This is clearly a highly relevant problem as humans have historically over-exploited ecosystems, or have done damage due to unforeseen network effects. Our example assumes a simple hunter-gatherer society, but in principle any ecosystem service (including the aforementioned pollination services for agriculture) can be modelled within P-graph. This feature is the basis for the use of “socio-” in the title. Interactions within human society are outside of the scope of this paper, but have been dealt with in earlier P-

graph papers. Our methodology provides a valuable contribution to the current set of tools for effective management of ecosystems as a resource base to sustain humans.

2.10. Per the statement about the solution of optimization problems on Line 340, the use of the term optimization implies maximizing a goal function with regard to costs. As such, this terminology inherently supports a perspective that overlooks the need for capacity and measures to facilitate resilience in the face of disturbance. Although the p-graph approach incorporates increased complexity of systems relative to many other network approaches by accommodating the analysis of different functions in a single network, it does not incorporate important aspects of ecosystems that aid in resilience, such as adaptation and evolutionary dynamics. As I already mentioned above with regard to the carnivore example, variables such as minimal viable population size, are essential to the prevention of species loss. Can these dimensions of ecosystem dynamics somehow be accounted for with the P-graph approach? If not, how can the results of the P-graph analyses be qualified in light of such information?

Response: Our criticality index provides a first step in establishing how P-graph can be used to deduce and then characterize ecosystems. Combinatorial feasibility of a network is a necessary, but not sufficient, condition to establish the true viability of the corresponding ecosystem. A new subsection entitled “Implications and Prospects” discusses the possible uses of P-graph methodology (both stand-alone and in conjunction with mainstream ENA tools) in the analysis of emergent properties of ecological networks. Ecosystem adaptation and dynamics, however, are beyond the scope of our present work. These extensions will be the subject of our future work.

P-graph can be used to perform a combinatorially complete search of ecological interactions to propose candidate ecological networks to represent a real ecosystem. The search procedure will automatically consider structural feasibility due to the ecological interactions. The resulting structures can serve as skeletal frameworks for subsequent detailed analysis that considers the magnitudes of the flows and interactions. By imposing constraints, the search algorithm allows us to compose feasible pathways.

:

In addition to its stand-alone capabilities, the P-graph framework can also be used in conjunction with other concepts and tools that are relevant to the effective management of ecosystems. Biodiversity loss and ecosystem collapse are major environmental issues; understanding emergent properties of ecological networks can provide valuable insights for retarding or stopping irreversible damage. The concept of resilience has become an important aspect in the design of different man-made networks which need to be designed to withstand shocks [32]. Resilient systems need to be planned to be able to absorb, recover from, and adapt to perturbations at the network level [33]. The concept can be extended to managing ecosystems to allow them to withstand pressure from climate change, human encroachment, and disruptive events such as natural disasters. In PNS problems, P-graph methodology has been shown to be effective for designing robust and resilient systems via process-level redundancy and network level degeneracy [34]. This capability can also be readily extended to PNS-like problems in ENA, so that rational and effective plans can be developed for the protection of ecosystems.
