# Supplementary material for: Socio-ecological network structures from process graphs
Source: PLoS One. 2020 Aug 4;15(8):e0232384. doi: 10.1371/journal.pone.0232384 (PMC7402476; doi:10.1371/journal.pone.0232384)
Supplement: Attachment — Submitted filename: Response to Reviewers 2.pdf [file pone.0232384.s006.pdf]

<u>POINT-BY-POINT RESPONSE</u>

(text in red pertains to the comments of the reviewers, the response is in black)

Comment from Reviewer 2:

1. The development and analysis of the Criticality Index is done in the Discussion. I suggest moving this to the Methods and Results sections.

   Response:
   We agreed with the reviewer and moved the introduction of the criticality index under the Methods section (see lines 219-240). Table 2 that contains the critical index first appears in the Discussion section, hence we kept the analysis of the critical index under the Discussion section.

2. On this read, I realized that there is some confusion in terminology. Consider clarifying:
   a. "Conventional ENA", which seems to refer to Ecosystem Network Analysis that developed from input-output analysis, should be made explicit as the term ecological network analysis refers to a broad array of both modeling and analyses.

      Response:
      We thank the reviewer for pointing this out, we have changed ENA to "ecological network analysis" that refers to a broader array of both modeling and analyses.

   b. P-graph the modeling and analysis technique and P-graph the software, sometimes referred to as P-graph Studio. I suggest making P-graph the software italicized or underlined, as titles often are.

      Response:
      The name of the P-graph software is "*P-graph Studio",* we followed the advice of the reviewer by italicizing "*P-graph Studio"*.

      > The P-graph software, called as *P-graph Studio*, is hosted and supported by the Department of Computer Science and Systems Technology at the University of Pannonia [24]. *P-graph Studio* can be accessed and used free of charge for research purposes via www.p-graph.org. It is currently on version 5.2.2.2 and can only run under Microsoft Windows. The following specifications are required: Microsoft .NET Framework 4.5.1 (x86 and x64) and Windows Installer 4.5. The website also contains a brief tutorial on P-graph fundamentals. Using the attached file in S1 Appendix, interested readers can replicate the case study that follows.
      >
      > Since *P-graph Studio* is designed for use on engineering design problems, this article comes with a prototype code implemented in Microsoft Excel and Visual Basic for Windows (VBA) which is more readily used for the analysis of social-ecological networks. The prototype is made freely available here and can be retrieved from S2 Appendix. Users can replicate the case study that follows, modify it, or even analyze new examples.
